# Synthesis of L-asparagine Catalyzed by a Novel Asparagine Synthase Coupled With an ATP Regeneration System

**DOI:** 10.3389/fbioe.2021.747404

**Published:** 2021-09-23

**Authors:** Wei Luo, Jinglong Xu, Huiying Chen, Huili Zhang, Peilong Yang, Xiaobin Yu

**Affiliations:** ^1^ The Key Laboratory of Carbohydrate Chemistry and Biotechnology, Ministry of Education, School of Biotechnology, Jiangnan University, Wuxi, China; ^2^ College of Chemistry and Bioengineering, Guilin University of Technology, Guilin, China; ^3^ College of Life Sciences, University of Shihezi, Shihezi, China; ^4^ Key Laboratory of Feed Biotechnology, Ministry of Agriculture and Rural Affairs, Institute of Feed Research of CAAS, Beijing, China

**Keywords:** l-Asparagine, asparagine synthase, class III polyphosphate kinase, ATP regeneration system, biocatalysis and biotransformation

## Abstract

Compared with low-yield extraction from plants and environmentally unfriendly chemical synthesis, biocatalysis by asparagine synthetase (AS) for preparation of L-asparagine (L-Asn) has become a potential synthetic method. However, low enzyme activity of AS and high cost of ATP in this reaction restricts the large-scale preparation of L-Asn by biocatalysis. In this study, gene mining strategy was used to search for novel AS with high enzyme activity by expressing them in *Escherichia coli* BL21 (DE3) or *Bacillus subtilis* WB600. The obtained *Lsa*AS-A was determined for its enzymatic properties and used for subsequent preparation of L-Asn. In order to reduce the use of ATP, a class III polyphosphate kinase 2 from *Deinococcus ficus* (*Dfi*PPK2-Ⅲ) was cloned and expressed in *E*. *coli* BL21 (DE3), Rosetta (DE3) or RosettagamiB (DE3) for ATP regeneration. A coupling reaction system including whole cells expressing *Lsa*AS-A and *Dfi*PPK2-Ⅲ was constructed to prepare L-Asn from L-aspartic acid (L-Asp). Batch catalytic experiments showed that sodium hexametaphosphate (>60 mmol L^−1^) and L-Asp (>100 mmol L^−1^) could inhibit the synthesis of L-Asn. Under fed-batch mode, L-Asn yield reached 90.15% with twice feeding of sodium hexametaphosphate. A final concentration of 218.26 mmol L^−1^ L-Asn with a yield of 64.19% was obtained when L-Asp and sodium hexametaphosphate were fed simultaneously.

## Introduction

As a common amino acid, L-asparagine (L-Asn) is widely used in medicine, food and other fields. L-Asn can be used as a precursor for some drugs (Bunev et al., 2014), a green catalyst or a precursor of synthetic catalysts (Garg et al., 2020; Safari et al., 2020), and also has antioxidant and other effects that is useful in food manufacturing (Echavarría et al., 2013). L-Asn can be prepared by a variety of synthetic methods, including chemical synthesis, extraction from plants and biosynthesis. Compared with the former two, biosynthesis has the advantages of simple production process, low equipment requirements, high production efficiency, low energy consumption and low pollution.

In plants, biosynthesis of L-Asn is derived from L-aspartic acid (L-Asp) by the catalysis of asparagine synthetase (AS) using NH^4+^ or glutamine as amide donor. The NH^4+^-dependent asparagine synthetase (AS-A) encoded by *asnA* gene only use NH^4+^ as an amide donor and exists in prokaryotes, while glutamine-dependent asparagine synthetase (AS-B) encoded by *asnB* gene can use either NH^4+^ or glutamine as an amide donor and widely exists in all kinds of organisms (Gaufichon et al., 2010).

Although AS has been successfully cloned and expressed (Kasai et al., 2000), research on the preparation of L-Asn by the catalysis of AS is limited. Due to the low activity of AS and the need to add expensive ATP, this reaction is not economically viable. Moreover, accumulation of by-products (AMP or ADP) during the reaction derived from the addition of ATP, which not only increases the difficulty of product separation and purification, but also causes product inhibition (Iwamoto et al., 2007). Therefore, it is necessary to build a cost-effective ATP regeneration system in order to overcome these problems.

The common way of ATP regeneration depend on kinase, such as acetate kinase (Yan et al., 2014), which has been used for the preparation of 3-phosphate glycerol (Li et al., 2001). Polyphosphate kinase (PPK) is another important one catalyzes reversible synthesis of ATP from ADP using polyphosphate as a phosphate donor and such ATP regeneration system has been used in the synthesis of d-allose (Xiao et al., 2019). However, a novel class III polyphosphokinase 2 (PPK2-Ⅲ) can catalyze both AMP and ADP phosphorylation to generate ATP (Motomura et al., 2014). Considering the simple operation (addition of a single enzyme), high availability and low cost of polyphosphate, PPK2-Ⅲ obviously represents as a more feasible candidate for ATP regeneration. Suzuki et al. (2018) co-expressed PPK2-Ⅲ and tyrosine synthase A in *Escherichia coli* BL21 (DE3) for whole-cell catalysis, in which 6.7 mmol mL^−1^ L-trp-L-pro was produced in 24 h with the addition of only 10 mmolmL^−1^AMP.

Here, AS with high enzyme activity was screened by gene mining strategy and *Lsa*AS from *Lactobacillus salivarius* was isolated. *Lsa*AS coupled with an ATP regeneration system using polyphosphokinase 2 from *D*. *ficus* (*Dfi*PPK2-III) was constructed in an one-pot whole-cell reaction for synthesis of L-Asn. The reaction conditions were investigated in order to obtain the optimal production of L-Asn.

## Materials and Methods

### Materials

The strains used here were purchased from the General Microorganisms Deposit Management Center of Jiangnan University (Supplementary Table S1). The tool plasmids used were previously deposited in our laboratory. The genes and primers were synthesized by Suzhou Jinweizhi Biotechnology Co., Ltd. Kits for genome extraction, plasmid extraction, DNA recovery and DNA purification were purchased from Shanghai Shenggong Co., Ltd. Restriction enzymes and DNA recombinases were obtained from Dalian TAKARA, Co., Ltd.

### Gene Mining of Asparagine Synthetase

For gene mining, AS-A and AS-B from *E. coli* were selected as probes (Wang et al., 2005), and BLAST procedures were conducted in the NCBI database to search for AS-A and AS-B with different sequence identities. In order to obtain enzymes with different enzymatic properties, representative proteins with different sequence identities were selected for gene cloning, expression and analyses of enzyme activities.

### Cloning and Expression of Asparagine Synthetase and Polyphosphokinase2-Ⅲ

Guided with the primers in Supplementary Table S2, different AS genes (*asn*) and polyphosphokinase 2 genes were amplified by polymerase chain reaction (PCR) using the genome of each strain as a template. Each *asn* was connected to pET28a and pMA5 and the recombinant plasmids were transformed into *E. coli* JM109 for gene cloning. Then the recombinant plasmids were extracted and transformed into *E. coli* BL21 (DE3) and *Bacillus subtilis* WB600, respectively. On the other hand, PPK2-Ⅲ genes from *Deinococcus ficus* and *D*. *phoenicis* were linked to pET21a by using DNA recombinase and transformed into *E. coli* strains.

The recombinant *E. coli* cells harboring expression plasmids were cultured in the TB liquid media supplementated with kanamycin (final concentration of 50 μg ml^−1^) until the optical density (OD_600_) reaching 0.4–0.6. Then isopropyl-β-d-Thiogalactopyranoside (IPTG) was added with a final concentration of 0.1 mmol L^−1^ and incubated at 16°C for 24 h. The recombinant *B. subtilis* WB600 cells harboring expression plasmids were cultured in the SB liquid media with kanamycin (final concentration of 50 μg ml^−1^) at 36°C for 24 h.

### Purification of Asparagine Synthetase and Polyphosphokinase2-Ⅲ

The collected cells were washed, resuspended in lysis buffer (20 mM Tris-HCl buffer, pH 8.0). Then cells were crushed by two rounds of ultrasound treatment and centrifuged at 12,000 rpm for 20 min at 4°C. Crude enzyme extract was prepared by filtering supernatant with a 0.22 μm microporous membrane for use. The crude extract was loaded onto a Ni-NTA column pre-equilibrated with buffer. The binding solution and eluent were used as the mobile phase for linear elution (eluent ratio: 0–100%), followed by SDS-PAGE analysis of the collected eluate.

### Whole-Cell Catalytic Preparation of L-Asn From L-Asp

The 5 ml reaction mixture contained 0.1 mol L^−1^ Tris-HCl (pH 8.0), 0.1–0.5 mol L^−1^ L-Asp, 0.1–0.8 mol L^−1^ NH_4_Cl, 1–10 mmol L^−1^ ATP, 5–250 mmol L^−1^ MgCl_2_, 5–150 mmol L^−1^ sodium hexametaphosphate and 1% Triton X-100. The reaction began with the addition of recombinant cells of AS and PPK2-Ⅲ. The reaction temperature is 25–45°C and the rotation speed is 180 rpm. Whole-cell catalytic reaction is terminated in a boiling water bath for 5 min.

### Assays of Asparagine Synthetase and Polyphosphokinase2-Ⅲ Activities

The activity of AS was measured in 1 ml reaction system containing 100 mmol L^−1^ Tris-HCl buffer (pH 8.0), 20 mmol L^−1^ L-Asp, 10 mmol L^−1^ MgCl_2_, 10 mmol L^−1^ NH_4_Cl (or L-Gln), 10 mmol L^−1^ ATP and 0.1 ml enzyme solution. After incubation at 37°C for 15 min, the sample was boiled for 5 min to terminate the reaction. Then the sample was centrifuged at 12,000 rpm and 4°C for 5 min, and the supernatant was filtered through a microporous (0.22 μm) membrane and poured into a sample bottle for content determination. The L-Asn content was determined by HPLC (High Performance Liquid Chromatography) with orthophthalaldehyde (OPA) on-line derivatization, and the amount of enzyme required to produce 1 μmol L-Asn from L-Asp per minute was defined as one enzyme activity unit.

The activity of PPK2-Ⅲ was measured in 1 ml reaction system containing 100 mmol L^−1^ Tris-HCl (pH 8.0), 10 mmol L^−1^ MgCl_2_, 50 mmol L^−1^ polyphosphate and 1 mmol L^−1^ AMP. The reaction was conducted at 37°C for 10 min, and the sample was heated at 80°C for 10 min to terminate the reaction. After centrifuge at 12,000 rpm for 10 min, the supernatant was collected to determine the ATP content by ATP detection kit. As for the definition of activity of PPK2-Ⅲ, the amount of enzyme required to produce 1 μmol of ATP per minute is defined as a unit of enzyme activity.

### HPLC Analyses of L-Asn, L-Asp and Glutamine

Concentrations of L-Asn, L-Asp and glutamine were determined by HPLC on-line o-phthalaldehyde (OPA)-derivation method with an Agilent Zorbax Eclipse AAA column (4.6 × 150 mm, 5 μm). The control conditions were set as follows, column temperature 40°C, detection wavelength 338 nm, flow rate 1.0 ml min^−1^, sample volume 10 μL. The mobile phase contained A (40 mmol L^−1^ Na_2_HPO_4_, pH 7.8) and B (acetonitrile: methanol: water = 45: 45: 10). The linear elution programme represented as the proportion of mobile phase B was as follows, 5% at 0–2 min, 5%–57% at 2–12 min, 100% at 13–17 min, 5% at 18–24 min.

## Results and Discussion

### Screening of Asparagine Synthetase

Through sequence alignment, the enzyme molecules exhibiting 20–100% identity with the amino acid sequence of the probe were selected in order to obtain a novel AS with different enzymatic property from the probe. Thereafter, eight genes of *asnB* and four genes of *asnA* were selected for clone and heterologous expression (Supplementary Table S1). Heterologous expression of these enzyme proteins in *E. coli* and *B. subtilis* is indicated by SDS-PAGE (Supplementary Figure S1 and Supplementary Figure S2). Theoretically, the molecular weights of AS-B and AS-A are ∼60 and ∼40 kDa, respectively. *Eco*AS-B, *Spl*AS-B, *Hal*AS-B and *Eco*AS-A were achieved high soluble protein expression and consistent with the theoretical value, while *Vru*AS-B, *Sma*AS-B had less soluble protein expression but consistent with the theoretical value in *E. coli*. *B*. *subtilis* is considered to be a good host of protein soluble expression and was selected as host for these enzyme proteins expression. SDS-PAGE shows that *Hal*AS-A, *Lsa*AS-A, *Eco*AS-B, *Spl*AS-B, *Hal*AS-B, *Vru*AS-B and *Sma*AS-B are expressed in soluble state in *B. subtilis* WB600 (Supplementary Figure S2). Enzyme activity assys in Table 1 shows *B. subtilis* is more suitable as a host for heterologous expression of these enzyme proteins, except for *Eco*AS-A. *Lsa*AS-A expressed in *B. subtilis* produced highest enzyme activity (1356.7 mU·mL^−1^) and this value increased to as high as 3850.0 mU·mL^−1^ under optimal culture condition in triangular flask.

**TABLE 1:** Activities of recombinant AS expressed in *E. coli* BL21 (DE3) and *B. subtilis* WB600.

Recombinant AS	Activity^a^/(mU·mL^−1^)	Activity^b^/(mU·mL^−1^)
*Eco*AS-B	149.2 ± 4.52	137.8 ± 7.25
*Spl*AS-B	58.4 ± 7.54	64.2 ± 4.88
*Hal*AS-B	172.5 ± 4.41	193.3 ± 12.72
*Vru*AS-B	ND^c^	27.5 ± 1.53
*Sma*AS-B	ND^c^	167.2 ± 25.24
*Eco*AS-A	851.9 ± 37.50	ND^c^
*Hal*AS-A	ND^c^	129.3 ± 64.87
*Lsa*AS-A	ND^c^	1356.7 ± 49.44

a The host was *E. coli* BL21 (DE3).

b The host was *B. subtilis* WB600.

c L-Asn was not detected.

### Enzymatic Properties of *Lsa*AS-A

**FIGURE 1:**
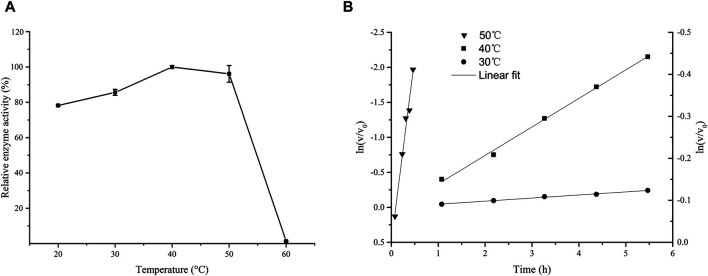
Effect of temperature on the activity of *Lsa*AS-A. **(A)** Optimal temperature of *Lsa*AS-A. **(B)** Plot of the first-order exponential decay formula to show the thermal stability of *Lsa*AS-A at 30, 40 and 50°C. The calculated values of the ln at 30°C and 40°C were calibrated by the left ordinate, while the counterpart at 50°C was calibrate by the right ordinate.

**FIGURE 2:**
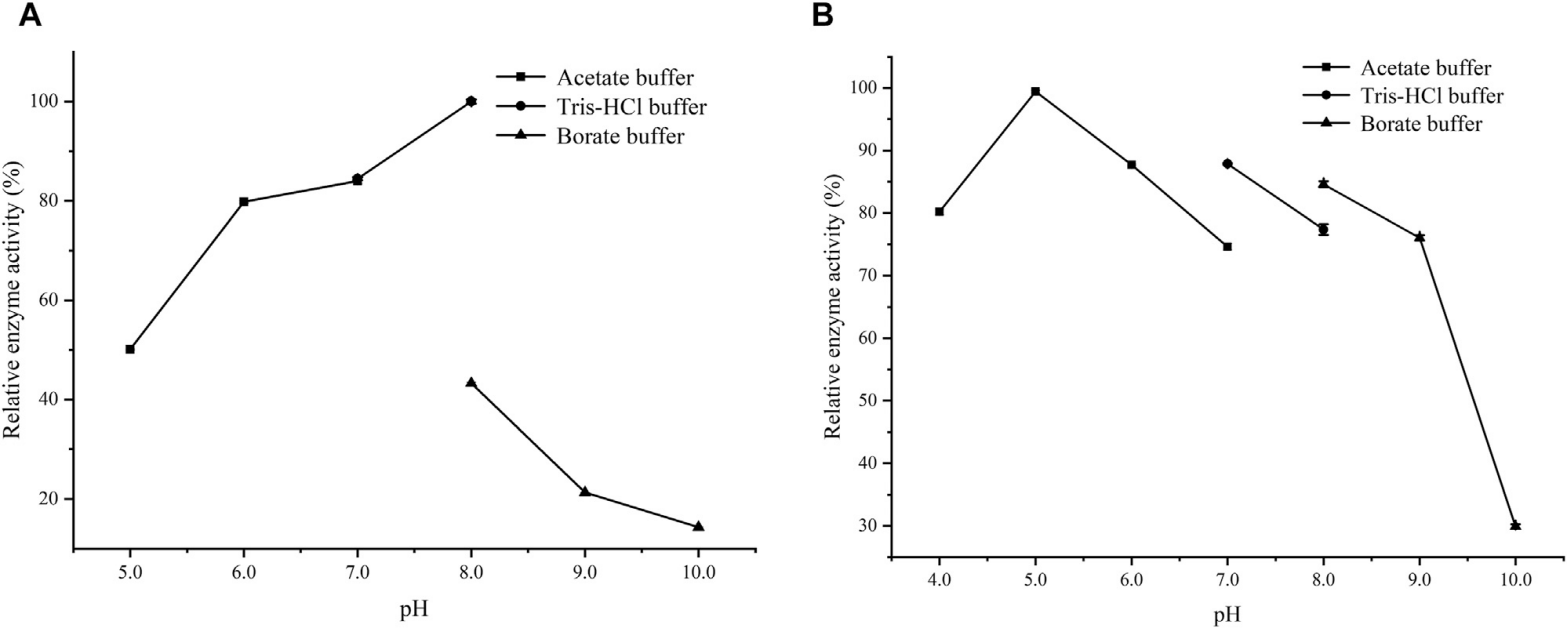
Effect of pH on the activity of *Lsa*AS-A. **(A)** Optimal pH of *Lsa*AS-A. **(B)** Stability of *Lsa*AS-A at different pH buffer solution.

Figure 1A shows that the activity of *Lsa*AS-A was temperature-dependent with an optimal temperature around 40°C. When temperature exceeded 50°C, the enzyme activity decreased rapidly. In order to obtain the half-life data of *Lsa*AS-A, the first-order exponential decay formula was used to determine the inactivation rate constant by plot of the decay formula (Figure 1B). Through calculation by inactivation rate constant, the half-lives of *Lsa*AS-A at 30, 40 and 50°C were 857.9, 94.6 and 51.0 min, respectively. In previous reports, the AS-A derived from *E. coli* had a half-life of less than 1 min at 45°C and was completely inactivated after being stored at 50°C for 5 min (Humbert and Simoni, 1980), suggesting that *Lsa*AS-A was significantly more stable than *E. coli*-derivived AS-A. Moreover, effect of pH on *Lsa*AS-A activity was also investigated by incubating the reaction solution under buffers with different pH. Figure 2A shows that *Lsa*AS-A is pH-dependent with an optimal pH of 8.0. The suitable reaction buffer seems to be acetate buffer and Tris-HCl buffer, rather than boric acid buffer. In addition, pH stability of *Lsa*AS-A was also investigated by storing *Lsa*AS-A at 4°C for 24 h under different pH conditions and its residual activity was measured. *Lsa*AS-A was stable in the acetate buffer at pH 5, while storage of *Lsa*AS-A in buffer with pH 4 or 6–9 resulted in partial loss of enzyme activity. When storage pH was up to 10, only 29.8% of the residual enzyme activity was retained (Figure 2B). On the other hand, metal ions were added to the reaction system to investigate the effect of them on enzyme activity and Table 2 shows that Mg^2+^, Mn^2+^, Fe^2+^, and Zn^2+^ have an activation effect on the enzyme activity. Among them, Fe^2+^ has the highest activation performance (136%), while Zn^2+^ gave a relatively low activation effect (23.61%). The lost of *Lsa*AS-A activity without addition of metal indicates that *Lsa*AS-A is a metal-dependent enzyme. The main reason is that metal ion including Mg^2+^ binds to the C-terminal active site of AS and participates in the reaction (Richards and Schuster, 1998).

**TABLE 2:** Effect of metal ions on the activity of *Lsa*AS-A.

Metal ion	Relative enzyme activity (%)
Control^a^	ND^b^
Na^+^	ND^b^
Ca^2+^	ND^b^
Cu^2+^	ND^b^
Fe^3+^	ND^b^
Mg^2+^	100
Mn^2+^	118.05 ± 0.47
Fe^2+^	136.36 ± 0.25
Zn^2+^	23.61 ± 0.13

a No metal ions were added.

b L-Asn was not detected.

In order to obtain K_m_ and V_max_, double reciprocal plot was used to determine these kinetic parameters and the results were shown in Table 3. The *K*_*m*_ of *Lsa*AS-A for Asp is 0.78 mmol L^−1^, which is similar to AS from *Klebsiella aerogenes* (Richards and Schuster, 1998), indicating that *Lsa*AS-A has a high affinity for Asp. The *K*_*m*_ of *Lsa*AS-A for NH_4_^+^ is 6.5 mmol L^−1^, which is similar to AS from *Streptococcus bovis* (Burchall et al., 1964). The *K*_*m*_ of *Lsa*AS-A for ATP is 2.24 mmol L^−1^, indicating that the affinity to ATP is high.

**TABLE 3:** Kinetic parameters of *Lsa*AS-A.

Substrate	*K*_m_ (mmol·L^−1^)	*V*_max_ (mmol·min^−1^·mL^−1^)
L-Asp	0.78	50.22
NH_4_^+^	6.5	53.76
ATP	2.24	46.00

### Cloning and Expression of Polyphosphokinase2-III and Preference for Polyphosphate With Different Degrees of Polymerization

PPK2-III from *Deinococcus ficus* and *D*. *phoenicis* was cloned and expressed in RosettagamiB (DE3), Rosetta (DE3) and BL21 (DE3) of *E. coli*, respectively. Electrophoretic analysis shows that they were expressed effectively (Supplementary Figure S3) and their enzyme activities were shown in Table 4. Each host can express ppk2-III, while *E. coli* Rosetta (DE3) exhibits the best expression performance among three hosts. The reason may be that *E. coli* Rosetta (DE3) can enhance the soluble expression of genes containing rare codons when compared with *E. coli* BL21 (DE3). Although *E. coli* RosettagamiB (DE3) also has this function, its slow growth restricts the improvement of enzyme activity. Considering that the polymerization degree has a great influence on the activity of most PPK (Ishige et al., 2002; Motomura et al., 2014; Meng et al., 2016), polyphosphates with different polymerization degrees were used to evaluate the activity of *Dfi*PPK2-Ⅲ. Among the measured polyphosphates, sodium hexametaphosphate and sodium polyphosphate could be used as substrates for *Dfi*PPK2-Ⅲ to synthesize ATP, while sodium pyrophosphate and sodium tripolyphosphates was not suitable as phosphate donors (Table 5).

**TABLE 4:** Enzyme activity of PPK2-III in different hosts.

Host	Enzyme activity (U·mL^−1^)	
	*Dfi*PPK2-III	*Dph*PPK2-III
*E. coli* RosettagamiB (DE3)	2.84 ± 0.15	3.04 ± 0.32
*E. coli* Rosetta (DE3)	13.19 ± 0.71	6.19 ± 0.42
*E. coli* BL21 (DE3)	7.89 ± 0.16	2.79 ± 0.08

**TABLE 5:** Effect of polyphosphate with different degrees of polymerization on the enzyme activity of *Dfi*PPK2-III.

Different polyphosphates	Activity (U·mL^−1^)
Sodium pyrophosphate	ND^a^
sodium tripolyphosphate	ND^a^
Hexametaphosphate	12.97 ± 0.35
Sodium polyphosphate mixture	0.52 ± 0.003

a Product was not be detected.

### Comparison of the Coupling Reaction System With Free Enzyme or Whole Cell

A coupling reaction system for L-Asn preparation was constructed using *Lsa*AS-A and *Dfi*PPK2-Ⅲ ATP regeneration system (Supplementary Figure S4) either including free enzymes or whole cells. From the point of view of application, free enzyme and whole cell have their own advantages and disadvantages. Table 6 shows that the catalysis efficiency of whole cell as a catalyst is higher than that of crude enzyme solution whether ATP or AMP was used as energy donor. The reason may be that endogenous PPase in living cell can eliminate the inhibitory effect of pyrophosphate (Suzuki et al., 2018; Lelièvre et al., 2020) and *Lsa*AS-A and *Dfi*PPK2-III remained in orininal cells were more stable than the free enzymes. Table 5 also indicates that the addition of surfactants to the whole-cell catalytic system can improve L-Asn titer, which may be ascribed to the fact that surfactant could enhance the permeability of the cell membrane and thereby improve the effect of mass transfer.

**TABLE 6:** Effect of different catalyst forms and energy donors on the production of L-Asn.

Catalyst form	L-Asn concentration (mmol·L^−1^)	
	ATP	AMP
Whole cell	5.83 ± 0.24	5.16 ± 0.04
Whole cell +1% Triton X-100	6.20 ± 0.32	5.72 ± 0.01
Enzyme	3.60 ± 0.06	2.60 ± 0.02

### Effects of Substrates and Co-factor on Whole Cell Catalytic Synthesis of L-Asn

**FIGURE 3:**
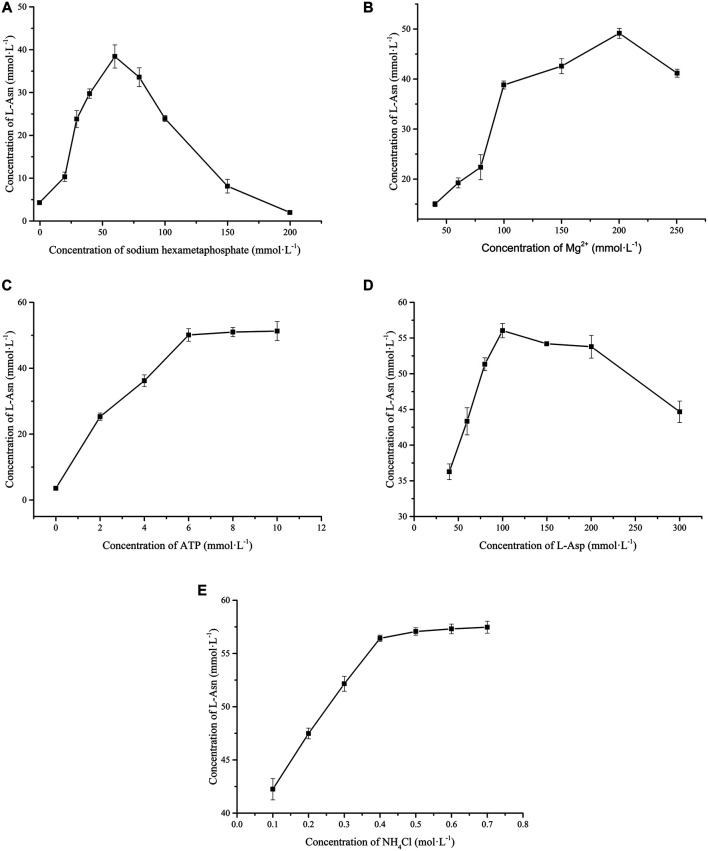
Effects of substrates and co-factor on whole cell catalytic preparation of L-Asn. **(A)** Sodium hexametaphosphate. **(B)** Mg^2+^. **(C)** ATP. **(D)** L-Asp. **(E)** NH_4_^+^.

Considering that polyphosphate is the phosphate donor for regeneration of ATP *via* the catalysis of PPK2-Ⅲ, the amount of polyphosphate added in the reaction system were investigated. Figure 3A shows that L-Asn production is hexametaphosphate-dependent. When the concentration of sodium hexametaphosphate added in the reaction solution was lower than 60 mmol L^−1^, the production of L-Asn increases with the enhancement of sodium hexametaphosphate concentration. While the concentration of sodium hexametaphosphate is greater than 60 mmol L^−1^, the production of L-Asn gave an opposite trend. The main reason may be that high concentration of sodium hexametaphosphate will chelate with Mg^2+^ to reduce enzyme activity (Iwamoto et al., 2007). Furthermore, sodium hexametaphosphate is loaded with high negative charge and is able to combine with enzyme, thereby reducing enzyme activity (Kameda et al., 2001).

As mentioned in enzymatic properties of *Lsa*AS-A, metal ions are necessary for *Lsa*AS synthesis of L-Asn, so DfiPPK2_S_ also requires Mn^2+^ or Mg^2+^ as cofactors (Sato et al., 2007; Motomura et al., 2014). However, the present study showed that Mn^2+^, Fe^2+^ and Zn^2+^ had no stimulating effect on L-Asn production (data not shown). The reason may be that their strong binding force with sodium hexametaphosphate leads to the formation of precipitation, so these ions can not bind to *Lsa*AS to play the role of cofactor (Larsen et al., 1999). As for Mg^2+^, Figure 3B shows that the situation is different. When the concentration of Mg^2+^ is no more than 200 mmol L^−1^, the production of L-Asn is stimulated by the enhancement of the concentration of Mg^2+^. However, the titer of L-Asn would be reduced if Mg^2+^ exceeds 200 mmol L^−1^ since chelation derived from high concentration Mg^2+^ is easy to genarate precipitation (Kameda et al., 2001).

In order to reduce costs, effect of initial ATP concentration (0–10 mmol L^−1^) on whole-cell catalytic synthesis of L-Asn was investigated. Without the addition of ATP, only 3.56 mmol L^−1^ of L-Asn was produced. When 6 mmol L^−1^ of ATP was added, the L-Asn output reached 50.10 mmol L^−1^ (Figure 3C). Theoretically, 1 mol ATP is consumed for every 1 mol L-Asn produced, so the ATP regeneration system has recycled ATP for 7.35 times. The final concentraion of L-Asn was not restricted by ATP when its intial concentraion was no less than 6 mmol L^−1^, so addition amount of 6 mmol L^−1^ is a suitable value in this ATP regeneration system.

Under the aforementioned optimal conditions, effects of L-Asp concentration (40–300 mmol L^−1^) and NH_4_^+^ concentration (0.1–0.7 mol L^−1^) on the whole-cell catalytic synthesis of L-Asn were investigated. When the concentration of L-Asp is 100 mmol L^−1^, the yield of L-Asn reaches the maximum (55.87 mmol L^−1^). L-Asp concentration high than 100 mmol L^−1^ would lead to reduction in L-Asn titer, which may be ascribed to substrate inhibition (Figure 3D). As for another substrate NH_4_^+^, Figure 3E shows that enhanced concentration of NH_4_^+^ improves the production of L-Asn, but 0.4 mol L^−1^ may be an optimal value since more addition of NH_4_^+^ is not able to bring about more production of L-Asn.

### Effect of Feeding Substrate on the Production of L-Asn

**FIGURE 4:**
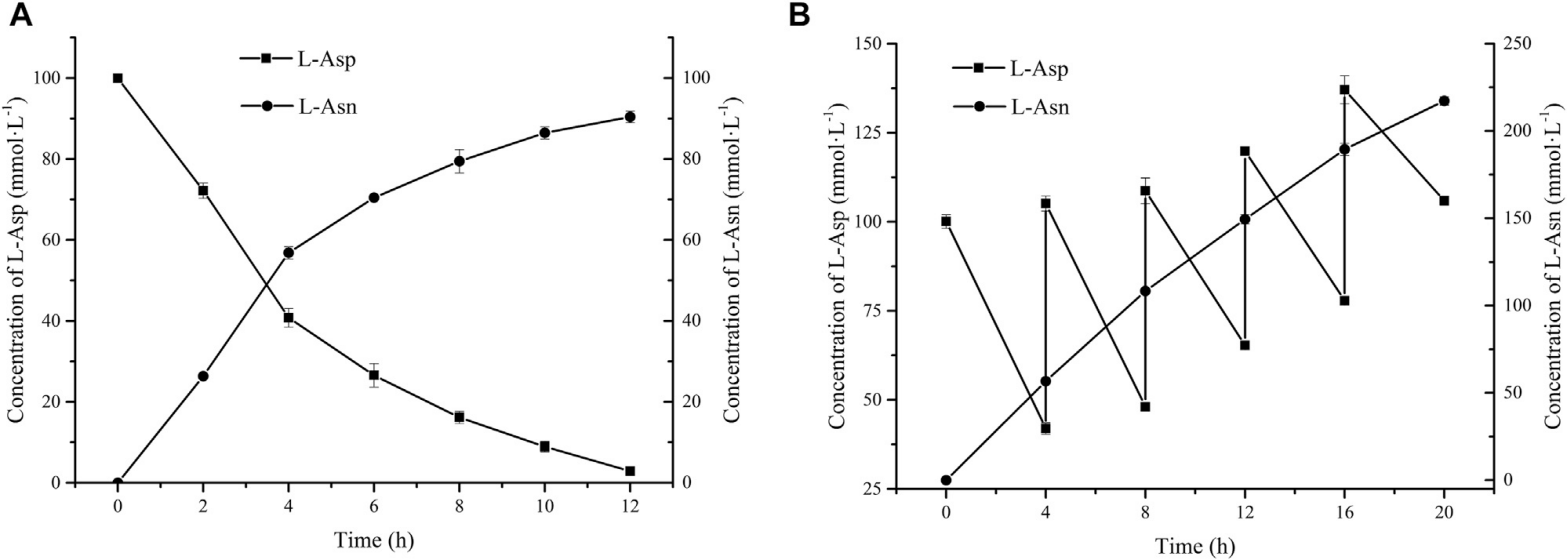
Effects of feeding substrates on whole cell catalytic preparation of L-Asn. **(A)** Sodium hexametaphosphate (60 mmol L^−1^) was added at 4 and 8 h, respectively. **(B)** Sodium hexametaphosphate (60 mmol L^−1^) and L-Asp (60 mmol L^−1^) were added at 4, 8, 12, 16, and 20 h, respectively.

Above results indicate that addition of high concentration of sodium hexametaphosphate will cause inhibition in L-Asn production due to its chelation of Mg^2+^ and inhibition of enzyme activity. In order to release the inhibitory effect of sodium hexametaphosphate, a fed-batch strategy for addition of sodium hexametaphosphate was used. After 12 h, the conversion rate reached 90.15% (Figure 4A), which was nearly 80% higher than that of adding 180 mmol L^−1^ sodium hexametaphosphate directly, indicating that feeding sodium hexametaphosphate in batches can suppress its negative effect (Kameda et al., 2001). Furthermore, the strategy of feeding L-Asp and sodium hexametaphosphate simultaneously was adopted in order to obtain higher yield of L-Asn. At 4, 8, 12, 16, and 20 h after the start of the reaction, L-Asp and sodium hexametaphosphate were fed at a final concentration of 60 mmol L^−1^. At the end of the catalytic reaction, 218.26 mmol L^−1^ L-Asn was produced with a yield of 64.19% (Figure 4B).

## Conclusion

Through gene mining, a novel asparagine synthetase *Lsa*AS-A with good catalytic performance was isolated. The optimal temperature and pH of *Lsa*AS-A was 41°C and 8.0, respectively; the half-life in the buffer solution at pH 8 and 30°C was 857.85 min. The enzyme was a metal-dependent enzyme and has a high affinity for L-Asp. In order to solve the problem of ATP supply in the reaction process, we expressed PPK2-III from *Deinococcus ficus* in *E*. *coli* Rosetta (DE3), which can use polyphosphate to synthesize ATP. A coupling catalytic reaction system including *Lsa*AS-A, *Dfi*PPK2-Ⅲ and substrate was then constructed to prepare L-Asn from L-Asp. Biotransformation experiments showed that the whole-cell reaction system could obtain higher product yield than free enzyme catalysis, so preparation of L-Asn from L-Asp by whole cell catalysis was optimized under batch modes. In order to reduce substrate inhibition, fed-batch strategy was used to control substrate concentration and 218.26 mmol L^−1^ L-Asn was obtained with a yield of 64.19%.

## Data Availability

The original contributions presented in the study are included in the article/Supplementary Material, further inquiries can be directed to the corresponding authors.
