# Supplementary material for: Synthesis of L-asparagine Catalyzed by a Novel Asparagine Synthase Coupled With an ATP Regeneration System
Source: Front Bioeng Biotechnol. 2021 Sep 23;9:747404. doi: 10.3389/fbioe.2021.747404 (PMC8495130; doi:10.3389/fbioe.2021.747404)
Supplement: Supplementary file 1 [file Table1.DOCX]

Table S1 Gene screening of asparagine synthetase

| No. | Gene | Enzyme | Source Strain | Identity to *Eco*AS |
| --- | --- | --- | --- | --- |
| 1 | *asnB* | *Eco*AS-B | *Escherichia coli* | 100% |
| 2 | *asnB* | *Spl*AS-B | *Serratia plymuthica* | 93% |
| 3 | *asnB* | *Hal*AS-B | *Hafnia alvei* | 86% |
| 4 | *asnB* | *Vru*AS-B | *Vibrio ruber* | 76% |
| 5 | *asnB* | *Sma*AS-B | *Stenotrophomonas maltophilia* | 60% |
| 6 | *asnB* | *Spa*AS-B | *Sphingomonas paucimobilis* | 56% |
| 7 | *asnB* | *Pex*AS-B | *Penicillium expansum* | 49% |
| 8 | *asnB* | *Bfi*AS-B | *Bacillus firmus* | 28% |
| 9 | *asnA* | *Eco*AS-A | *Escherichia coli* | 100% |
| 10 | *asnA* | *Hal*AS-A | *Hafnia alvei* | 77% |
| 11 | *asnA* | *Pma*AS-A | *Pseudoalteromonas marina* | 69% |
| 12 | *asnA* | *Lsa*AS-A | *Lactobacillus salivarius* | 53% |

Table S2 Primers used in this study

| Primer name | Primer sequence |
| --- | --- |
| *Eco*AS-B-pET28a-F | ATGGGTCGCGGATCCGAATTCATGTGTTCAATTTTTGGCGTATTC |
| *Eco*AS-B-pET28a-R | CTCGAGTGCGGCCGCAAGCTTTTACTTATATGCCGACTGGTGAACA |
| *Spl*AS-B-pET28a-F | CAGCAAATGGGTCGCGGATCCATGTGTTCTATTTTCGGTGTGCTC |
| *Spl*AS-B-pET28a-R | CTCGAGTGCGGCCGCAAGCTTTTACTTGTAGGCGGATTGGTGG |
| *Hal*AS-B-pET28a-F | CAGCAAATGGGTCGCGGATCCATGTGTTCTATATTTGGCATCTTAGATATAA |
| *Hal*AS-B-pET28a-R | GCAAGCTTGTCGACGGTGCACTTAATAAGCTTCCTGATGAACGTTTT |
| *Vru*AS-B-pET28a-F | CAGCAAATGGGTCGCGGATCCATGTGTTCTATATTTGGCATCTTAGATATAA |
| *Vru*AS-B-pET28a-R | GCAAGCTTGTCGACGGTCGACTTAATAAGCTTCCTGATGAACGTTTT |
| *Sma*AS-B-pET28a-F | CAGCAAATGGGTCGCGGATCCATGTGTTCGATCTTCGGAATCTTC |
| *Sma*AS-B-pET28a-R | GCAAGCTTGTCGACGGTCGACTTAGGCCAGTGCCTGCTCG |
| *Spa*AS-B-pET28a-F | CAGCAAATGGGTCGCGGATCCATGTGCGGTATCGCCGGC |
| *Spa*AS-B-pET28a-R | CTCGAGTGCGGCCGCAAGCTTTCAGAACAGCCGCTTCAGGC |
| *Bfi*AS-B-pET28a-F | CAGCAAATGGGTCGCGGATCCATGTGTGGAATTACCGGCTGG |
| *Bfi*AS-B-pET28a-R | GCAAGCTTGTCGACGGTCGACTCATTCTGCAATATTTATATTATAATCCTTAA |
| *Eco*AS-A-pET28a-F | ATGGGTCGCGGATCCGAATTCATGTGTTCAATTTTTGGCGTATTC |
| *Eco*AS-A-pET28a-R | CTCGAGTGCGGCCGCAAGCTTTTACTTATATGCCGACTGGTGAACA |
| *Hal*AS-A-pET28a-F | CAGCAAATGGGTCGCGGATCCATGAAAAAACAATTTATACAAAAACAACA |
| *Hal*AS-A-pET28a-R | GCAAGCTTGTCGACGGTCGACTTAGAGAAGCGAGTCAATTTGATCG |
| *Pma*AS-A-pET28a-F | CAGCAAATGGGTCGCGGATCCATGAGTTCACACTATGTGCAGCAGC |
| *Pma*AS-A-pET28a-R | TGCGGCCGCAAGCTTGTCGACTTAGAGTAGACCGTCAACGCTTGC |
| *Lsa*AS-A-pET28a-F | ATGGGTCGCGGATCCGAATTCTTGGATTTAATTATTCCAAAGGATTATG |
| *Lsa*AS-A-pET28a-R | CTCGAGTGCGGCCGCAAGCTTCTACAAAATATGAATACCATTTTCTTCACA |
| *Eco*AS-B-pMA5-F | AAAGTGAAATCAGGGGGATCCATGTGTTCAATTTTTGGCGTATTC |
| *Eco*AS-B-pMA5-R | TCTGGTACGTACCAAGCTAGCTTACTTATATGCCGACTGGTGAACA |
| *Spl*AS-B-pMA5-F | AAAGTGAAATCAGGGGGATCCATGTGTTCTATTTTCGGTGTGCTC |
| *Spl*AS-B-pMA5a-R | TCTGGTACGTACCAAGCTAGCTTACTTGTAGGCGGATTGGTGG |
| *Hal*AS-B-pMA5-F | AAAGTGAAATCAGGGGGATCCATGTGTTCTA TCTTTGGTGT GCTTG |
| *Hal*AS-B-pMA5-R | TCTGGTACGTACCAAGCTAGC CTACTTTTGA TAAGCCGACT GATGTAC |
| *Vru*AS-B-pMA5-F | AAAGTGAAATCAGGGGGATCCATGTGTTCTATATTTGGCATCTTAGATATAA |
| *Vru*AS-B-pMA5-R | TCTGGTACGTACCAAGCTAGCTTAATAAGCTTCCTGATGAACGTTTT |
| *Sma*AS-B-pMA5-F | AAAGTGAAATCAGGGGGATCCATGTGTTCGATCTTCGGAATCTTC |
| *Sma*AS-B-pMA5-R | TCTGGTACGTACCAAGCTAGCTTAGGCCAGTGCCTGCTCG |
| *SpaAS-B-pMA5-F* | AAAGTGAAATCAGGGGGATCCATGTGCGGTATCGCCGGC |

Continued Table S2 Primers used in this study

| Primer name | Primer sequence |
| --- | --- |
| *SpaAS-B-pMA5-R* | TCTGGTACGTACCAAGCTAGCTCAGAACAGCCGCTTCAGGC |
| *Bfi*AS-B-pMA5-F | AAAGTGAAATCAGGGGGATCCATGTGTGGCA TTTTTGCTTG |
| *Bfi*AS-B-pMA5-R | TCTGGTACGTACCAAGCTAGCTAATATTTC TGATTATGAA TGGCG |
| *Pex*AS-B-pMA5-F | TAGAGTCGAGCTCAAGCTAGCATGTGTTCAATTTTTGGCGTATTC |
| *Pex*AS-B-pMA5-R | ATTTCGACCTCTAGAACGCGTTTACTTATATGCCGACTGGTGAACA |
| *Eco*AS-A-pMA5-F | TAGAGTCGAGCTCAAGCTAGCATGAAAACCGCTTACATTGCC |
| *Eco*AS-A-pMA5-R | TCTGGTACGTACCAAGCTAGCTTACAGCAGAGAAGGGACGTTC |
| *Hal*AS-A-pMA5-F | AAAGTGAAATCAGGGGGATCCATGAAAAAACAATTTATACAAAAACAACA |
| *Hal*AS-A-pMA5-R | TCTGGTACGTACCAAGCTAGCTTAATGATGATGATGATGATGGAGAAGCGAGTCAATTTGATCG |
| *Pma*AS-A-pMA5-F | AAAGTGAAATCAGGGGGATCCATGAGTTCACACTATGTGCAGCAGC |
| *Pma*AS-A-pMA5-R | TCTGGTACGTACCAAGCTAGCTTAGAGTAGACCGTCAACGCTTGC |
| *Lsa*AS-A-pMA5-F | AAAGTGAAATCAGGGGGATCCTTGGATTTAATTATTCCAAAGGATTATG |
| *Lsa*AS-A-pMA5-R | TCTGGTACGTACCAAGCTAGCCTAATGATGATGATGATGATGCAAAATATGAATACCATTTTCTTCACA |
| *Dei*PPK2s-pET21a-F | ACAGCAAATGGGTCGGGATCCATGAATTCCAAAGCATCACAGCA |
| *Dei*PPK2s-pET21a-R | TGGTGGTGCTCGAGTGCGGCCGCTTACCTGCTGGGAATATCCTCAA |
| *Dfi*PPK2s-pET21a-F | ACAGCAAATGGGTCGGGATCCATGAAAACCGACCGGTACCG |
| *Dfi*PPK2s-pET21a-R | TGGTGGTGCTCGAGTGCGGCCGCGATGCGGACTTCCTTGGGG |
| *DphPPK2s-pET21a-F* | ACAGCAAATGGGTCGGGATCCATGAACCCGGATCCGTACCG |
| *DphPPK2s-pET21a-R* | TGGTGGTGCTCGAGTGCGGCCGCGATTTTCAGCGTGGCGGC |

The underlined suquences of base are the enzyme digestion sites of specific tool enzymes. GAATTC (*Eco*R I), AAGCTT (*Hin*d III), GGATCC (*Bam*H I),GTCGAC (*Sal* I),GCTAGC (*Nhe* I), GCGGCCGC (*Not* I).

（A）


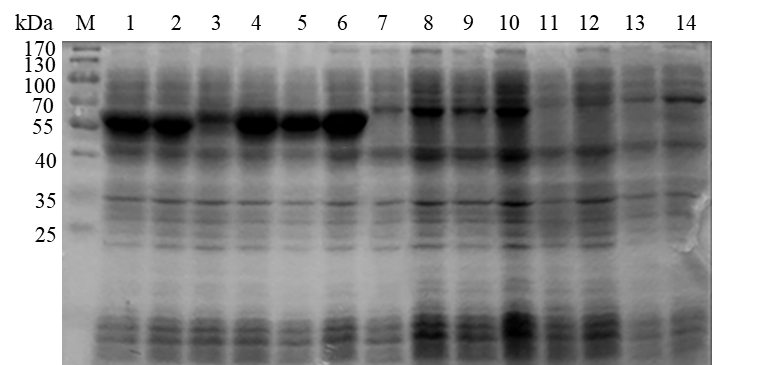


（B）


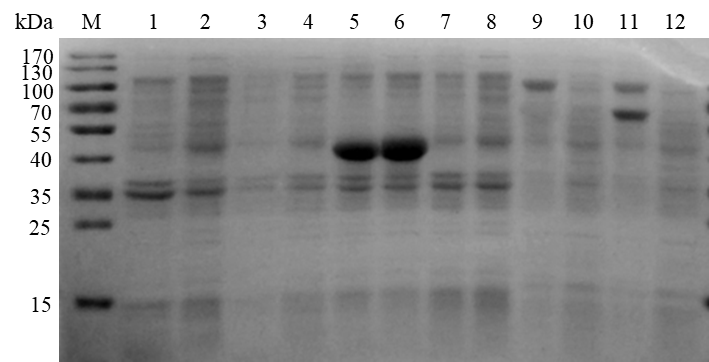


**Figure S1.** SDS-PAGE of asparagine synthetase expressed in [recombinant](javascript:;) *E.* [*coli*](javascript:;) BL21(DE3)

M, protein marker; Odd sequence numbers refer to supernatant of recombinant *E*. *coli* crushing solution, and even sequence numbers refer to precipitation of recombinant *E*. *coli* crushing solution. (A) 1 and 2, *Eco*AS-B; 3 and 4, *Spl*AS-B; 5 and 6, *Hal*AS-B; 7 and 8, *Vru*AS-B; 9 and 10, *Sma*AS-B; 11 and 12, *Spa*AS-B; 13 and 14, *Pex*AS-A; (B) 1 and 2, *Bfi*AS-B; 3 and 4, *Hal*AS-A; 5 and 6, *Eco*AS-A; 7 and 8, *Pma*AS-A; 9 and 10, *Lsa*AS-A.

（A）


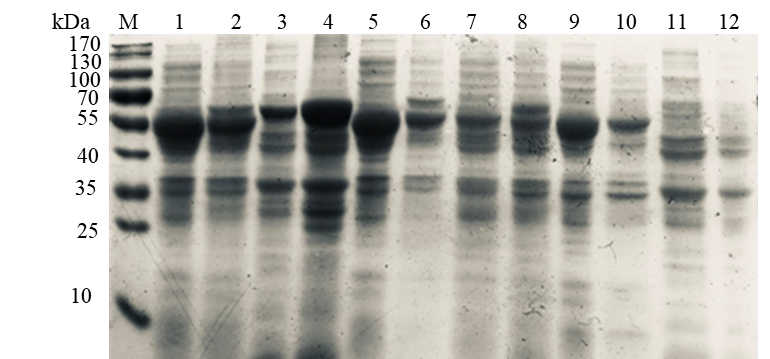


（B）


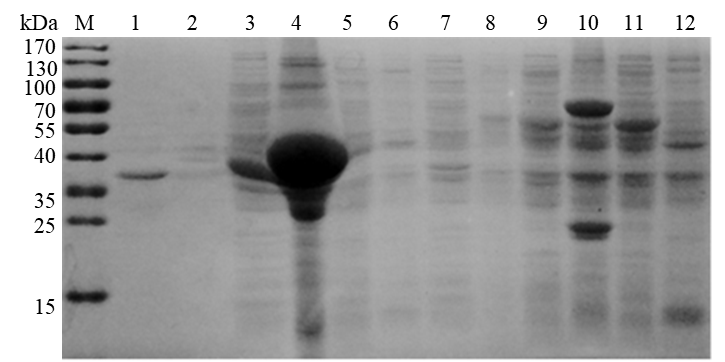


**Figure S2.** SDS-PAGE of asparagine synthetase expressed in [recombinant](javascript:;) *B. subtilis* WB600

M, protein marker; Odd sequence numbers refer to supernatant of recombinant *B. subtilis* crushing solution, and even sequence numbers refer to precipitation of recombinant *B. subtilis* crushing solution. (A) 1 and 2, *Eco*AS-B; 3 and 4, *Spl*AS-B; 5 and 6, *Hal*AS-B; 7 and 8, *Vru*AS-B; 9 and 10, *Sma*AS-B; 11 and 12, *Spa*AS-B; (B) 1 and 2, *Lsa*AS-A; 3 and 4, *Hal*AS-A; 5 and 6, *Pex*AS-A; 7 and 8, *Eco*AS-A; 9 and 10, *Pex*AS-A; 11 and 12, *Bfi*AS-B.


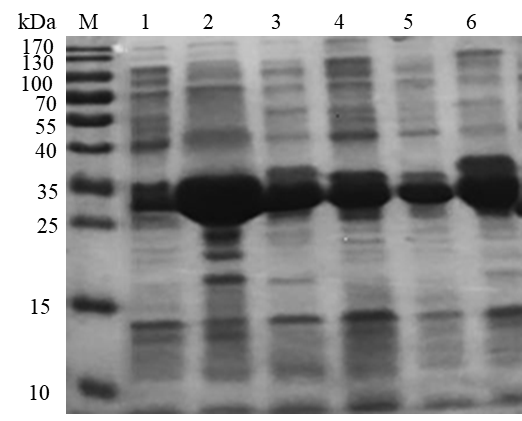


**Figure S3.** SDS-PAGE of PPK2-III expressed in different hosts

M, marker; 1, supernatant of Rosettagamib (DE3)/pET21a-*Dfi*ppk2 crushing solution; 2, supernatant of Rosetta(DE3)/pET21a-*Dfi*ppk2 crushing solution; 3, supernatant of BL21 (DE3)/pET21a*-Dfi*ppk2 crushing solution; 4, supernatant of Rosettagamib (DE3)/pET21a-*Dph*ppk2 crushing solution; 5, supernatant of Rosetta(DE3)/ pET21a-*Dph*ppk2 crushing solution; 6, supernatant of BL21(DE3)/ pET21a-*Dph*ppk2 crushing solution.


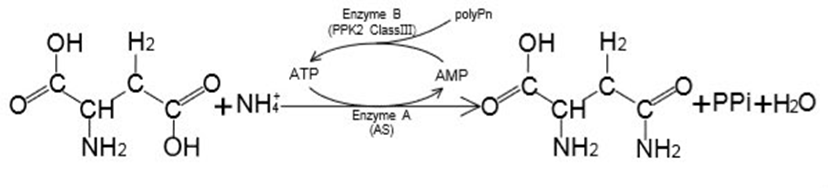


**Figure S4.** Schematic diagram of L-Asn synthesis based on asparagine synthase and class III PPK2 ATP regeneration system
